# Downregulation of calcineurin activity in cervical carcinoma

**DOI:** 10.1186/1475-2867-5-7

**Published:** 2005-04-01

**Authors:** S Padma, A Pavani Sowjanya, Usha Rani Poli, Meenakshi Jain, BN Rao, Gayatri Ramakrishna

**Affiliations:** 1 Centre for DNA Fingerprinting and Diagnostics, Nacharam, Hyderabad, A.P, India; 2 M.N.J Institute of Oncology and Regional cancer centre, Hyderabad, A.P, India; 3 Mediciti Hospital, Medchal, A.P, India

**Keywords:** cervical neoplasia, squamous cell carcinoma (SCC), calcineurin (PP2B/CaN), calmodulin (CaM)

## Abstract

**Background:**

Calcineurin (CaN) is an important serine-threonine phosphatase (PP2B), which plays a crucial role in calcium-calmodulin mediated signal transduction events. Calcineurin has been implicated in pathogenesis of various diseases cardiac hypertrophy, diabetic neuropathy and Alzheimer's, however its role in neoplasia remains unclear.

**Results:**

In view of this we evaluated the calcineurin activity in serum and biopsy samples collected from women diagnosed with invasive squamous cell carcinoma of cervix. A significant reduction was observed in the calcineurin activity in cancer cervix patients compared to the control group. However the calcineurin activity remained unaltered in the cervical scrapes obtained from patients diagnosed with low-grade squamous intra epithelial lesions (LSIL). Interestingly the downregulation of calcineurin activity in squamous cell carcinomas was not accompanied by any significant change in DNA-binding affinity of the transcriptional factor NFAT (Nuclear Factor of Activated T-cells). All the squamous cell carcinoma samples used in the present study were positive for high-risk human papillomavirus (HPV) types.

**Conclusion:**

The present study demonstrates the downregulation of calcineurin activity in squamous cell carcinoma of cervix with high risk HPV infection. We conclude that perturbations in calcineurin-mediated pathway may be involved in development of cervical neoplasia.

## Introduction

Cervical cancer is one of the most prevalent neoplastic diseases affecting women world wide, especially in the developing countries [1,2]. In India, cervical neoplasia is not only the most common malignancy but also a major cause of death among middle-aged women particularly in the rural areas [3,4]. It is well established now that infection with human papilloma virus (HPV) is a major etiological risk factor for development of cervical cancer, however other host/environmental factors contribute to the progression of the disease [5-7]. Infection with HPV can modulate the host factors involved in signaling cascades and deregulation of signaling pathways is a hallmark of cancer. An important feature of the signal transduction pathway is reversible serine/threonine phosphorylation of proteins and any aberrant activity in the kinases or phosphatases can disturb the phosphorylation/dephosphorylation cycle leading to uncontrolled proliferation. Therefore, identification of key-signaling molecules (kinases and phosphatases) responsible for neoplastic development can have potential clinical relevance allowing for diagnosis, early detection and intervention.

There is a plethora of information existing on the involvement of various kinases such MAPK [8], PI3K [9], JNK [10], cdk4 [11,12], cdc2 [13], Her2 [14], p38 [15] etc. in cervical carcinoma cells. While the phosphatases such as cdc25A/C and PTEN have been shown to be associated with cervical cancer [12,16,17], the existing information falls short on the involvement of serine/threonine protein phosphatase family members viz., PP1, PP2A, PP2B (calcineurin) and PP2C. There is only one report, which demonstrates downregulation of protein phosphatase 1, regulatory subunit in cervical cancer cells [18]. In view of the limited information available on involvement of serine/threonine phosphatase, an attempt was made in the present study to investigate if there is any association between calcineurin and cervical neoplasia.

Calcineurin is the only serine/threonine phosphatase whose activity is modulated by both calcium and calmodulin and it is a crucial mediator of the calcium mediated signal transduction pathways. Calcineurin is a heterodimeric protein consisting of a catalytic subunit (calcineurin A), with an active metal binding centre, and a regulatory subunit (calcineurin B), which binds to calcium [19]. One of the major functions of calcineurin is T-cell activation by dephosphorylation of the transcriptional factor NFAT (Nuclear Factor of Activated T-cells) [20]. Calcineurin is involved in a wide variety of biological responses including neuronal and muscle development, neurite outgrowth, morphogenesis of vertebrate heart valves, memory development, behavioral response etc [21-26]. In addition, calcineurin has also been implicated in pathogenesis of diseases such as cardiac hypertrophy, Alzheimer's, schizophrenia, diabetic nephropathy and Down's syndrome [27-32]. Calcineurin plays a role in apoptosis via inducing the cytochrome c/caspase dependent pathway and by dephosphorylating Bad, a proapototic member of Bcl-2 family [33,34]. Recent studies have also demonstrated the involvement of calcineurin in the cell cycle by regulation of the cdk4 (cyclin dependent kinase 4), a G_0_/G_1 _checkpoint element [35]. The immunosuppressant cyclosporin A (CsA), which specifically inhibits calcineurin activity, has proved to be a useful tool in understanding the role of calcineurin in various cellular processes. CsA has been shown to exert an antiproliferative effect not only in T cell but also in a wide variety of cells including lymphomas, keratinocytes, fibroblasts and smooth muscle cells indicating that calcineurin activity is important for cell cycle progression [36]. In contrast to the antiproliferative action of CsA in cell culture system, it has been shown that treatment with CsA in kidney transplant recipients is associated with increased incidence of renal cancer [37,38]. However it is still unclear if the genesis of cancer in CsA administered persons is due to direct inhibition of calcineurin-mediated pathways or its other indirect effects. This warrants a study on involvement of calcineurin in cancer progression and therefore the present study was undertaken to ascertain the importance of calcineurin in cervical neoplasia.

## Materials and methods

### Specimen collection

For the present study patient's consent was taken and the study was approved by the institutional bioethical committee. Cervical biopsy samples were collected (N = 45) from women diagnosed with invasive cervical cancer. The biopsy samples collected from patients were immediately snap frozen in liquid nitrogen. Cervical tissue specimens (N = 30) collected from women undergoing hysterectomy for non-neoplastic conditions formed the control group. Blood samples (3 ml) were collected in plain bottles [without anticoagulant] from both the normal and cancer patients. Blood was allowed to clot and serum was separated by centrifugation at 3000 rpm for 10 min at room temperature. The serum samples were stored at -20°C till further use.

Cervical scrapes were also collected using Ayer's spatula from women attending the cervical screening outpatient clinic (N = 30). Four of these women were found to have low-grade intraepithelial lesion (LSIL) and the rest with chronic inflammation only on cytology. The cervical scrape was collected in chilled phosphate buffer saline (pH 7.2) and the cells were collected by centrifugation at 13000 rpm for 10 min at 4°C. The supernatant was discarded and the cells were immediately frozen in liquid nitrogen till further use.

### Sample preparation

Tissues (100 mg) and cervical scrapes were homogenized in 500 μl or 250 μl of buffer A respectively, containing 25 mM HEPES (pH 7.2), 150 mM NaCl, 1% NP-40, 10 mM MgCl_2_, 10% glycerol, 10 μg/ml PMSF and 10 μg/ml aprotinin for 3 min. The homogenates were cleared of the debris by centrifugation at 13,000 rpm for 10 min at 4°C. The supernatants were aliquoted and stored at -70°C till further use.

### Assay for calcineurin

An aliquot of the homogenized sample (100 μl) was passed through sephadex G-25 column (Amersham Pharmacia) to eliminate the free phosphate and further used for calcineurin activity. The protein estimation was done using the BCA Kit [Pierce company] as per the manufacturers protocol. Calcineurin activity was assayed in a total volume of 50 μl containing 25 μl of 2X assay buffer (200 mM NaCl, 100 mM Tris [pH 7.5], 12 mM MgCl_2_, 1 mM CaCl_2_) and 5 μl of the cell/tissue homogenate. The samples were incubated with 2X-assay buffer for 10 min at 30°C. The total volume was adjusted to 50 μl by the addition of water. Reactions were initiated by adding RII phosphopeptide [5 μM] and incubated for a further period of 10 min at 30°C. Reactions were terminated by addition of 100 μl of Malachite green reagent (3 vol. of 0.045% Malachite green and 1 vol. of 4.2% ammonium molybdate in 4N HCl). The color was allowed to develop for 30 min and the absorbance read at 660 nm. The calcineurin activity was calculated as nmoles of inorganic phosphate released/min/mg total protein in case of tissues and for serum samples as nmoles of inorganic phosphate released/min/dl of serum.

### Determination of calmodulin and calcineurin contents in tissue lysates

The calmodulin and calcineurin contents were determined by competitive ELISA methods as described earlier [39]. The calcineurin antibody against the catalytic subunit A was used in this study.

### Electrophoretic mobility shift assay for NFATc-DNA binding

Electrophoretic mobility shift assay (EMSA) was performed using oligonucleotide with consensus binding site for NFATc (5'CGCCCAAAGAAAATTTGTTTCATA3'). Briefly, biopsy tissues were homogenized and the nuclear pellet was collected by centrifugation (600 × g: 10 min, 4°C). The nuclear lysates (100 μg) were prepared and incubated in 25 μl of reaction mixture containing polydI/dC, 10X binding buffer (50 μM ZnCl _2,_0.25 mM DTT, 20 mM Tris [pH 7.5], 60 mM KCl, 1 mM MgCl_2 _0.1 mM EDTA, 10% glycerol) and the labeled oligo for 30 min at room temperature. The reactions were terminated using 25 μl of the loading dye and loaded onto a 7% gel retardation assay gel. The run was done at 250 V for 3 hrs at 4°C. At the end of the run, the gel was removed, dried and exposed in a phosphoimager overnight at room temperature.

### HPV-testing and Typing

The DNA isolated from the squamous cell carcinoma samples were tested for presence of HPV by a PCR based reverse line blot assay as described earlier (40)

### Statistical analysis

Comparisons between the normal and the cancer samples were done using the Student's *t*-test for paired data.

## Results

### 1. Calcineurin activity in serum and cervical tissue (normal vs squamous cell carcinoma)

**Figure 1:**
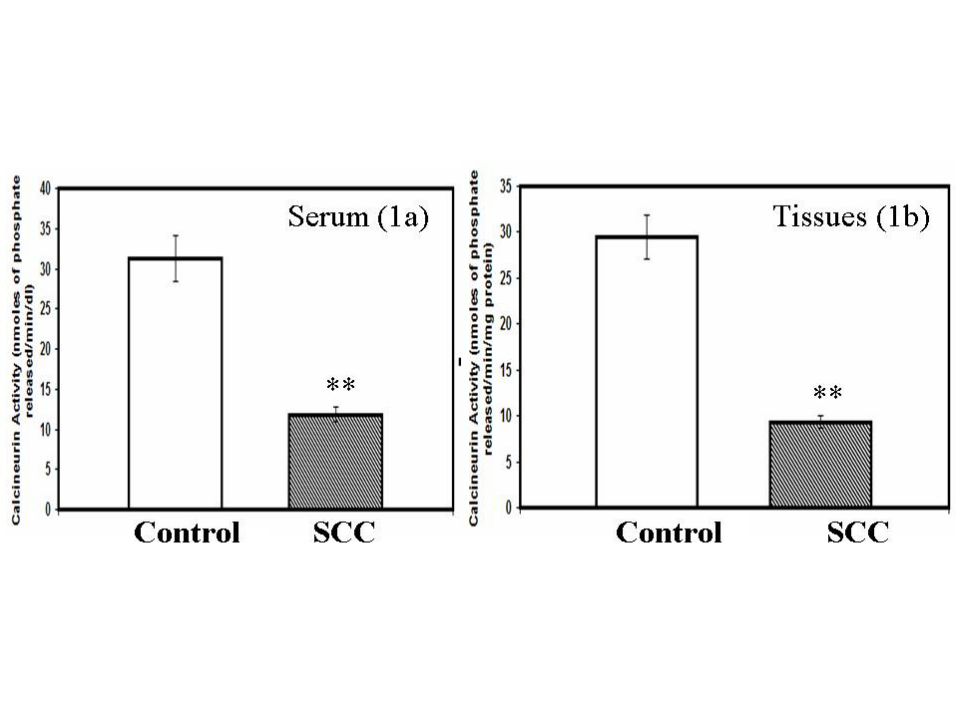
Calcineurin activity in serum (a) and biopsy samples (b) of control and cervical cancer patients diagnosed with squamous cell carcinoma (SCC). The calcineurin activity is significantly reduced in serum samples [1a, p = 0.0001] and in tissue lysates [1b, p = 0.0037] obtained from cancer patients compared to the normal group. The calcineurin activity was calculated as nmoles of inorganic phosphate released per mg protein of tissues or per deciliter of serum.

Calcineurin activity was assayed in both sera and tissue lysate by measuring the dephosphorylation of RII peptide, a specific substrate of calcineurin. The biopsy samples collected from the cancer patients were classified as moderately differentiated large cell non-keratinising squamous cell carcinomas of the cervix. We observed a significant reduction in the calcineurin activity both in the cervical tissues (p = 0.0001) and sera (p = 0.0037) of patients with invasive cervical cancer in comparison to the control group (Fig. 1). It has to be noted that we have calculated the calcineurin activity in serum as nmoles of phosphate released/min/deciliter. However if we consider the total protein content per deciliter of serum for each sample and accordingly calculate the calcineurin activity per milligram of serum protein, the calcineurin activity in serum will be much lower than that of tissue. Since the international standard for representing the units for enzyme activity for serum/plasma is represented in units per deciliter/litre the same has been represented in Fig. 1a.

### 2. Calmodulin and calcineurin A content in cervical tissues (normal vs squamous cell carcinoma)

**Figure 2:**
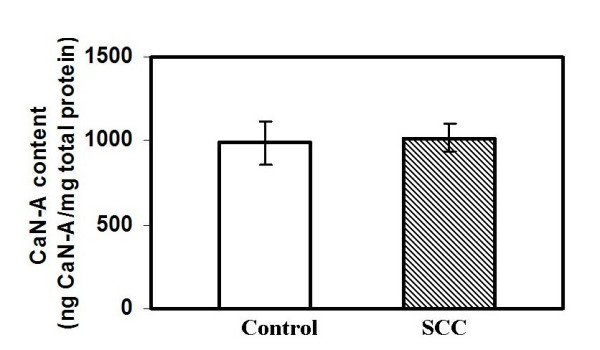
Total calcineurin content in normal and cervical cancer tissue lysates [SCC]. The calcineurin content was assayed by using antibody specific to calcineurin [Sigma monoclonal anti-CaNα, 1:7500 dilution using a sandwich ELISA method.

**Figure 3:**
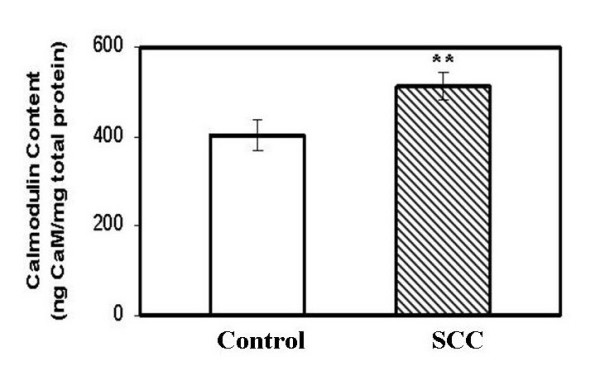
Total calmodulin content in normal and cervical cancer tissue lysates (SCC) were determined by using antibody specific to calmodulin. Note a significant increase [p = 0.0263] in calmodulin content in cancer patients.

The calmodulin and calcineurin contents were also analyzed in the biopsies, to evaluate if the decrease in the calcineurin activity could be correlated with their respective contents. The content for both calcineurin A subunit and calmodulin was measured using competitive ELISA method in the tissue samples. There was no change in the level of calcineurin A between the control and the cancer samples (Fig. 2). Interestingly, there was a significant increase in the calmodulin level in cervical carcinoma (Fig. 3).

### 3. Calcineurin activity in Low-grade squamous cell intraepithelial lesions

**Figure 4:**
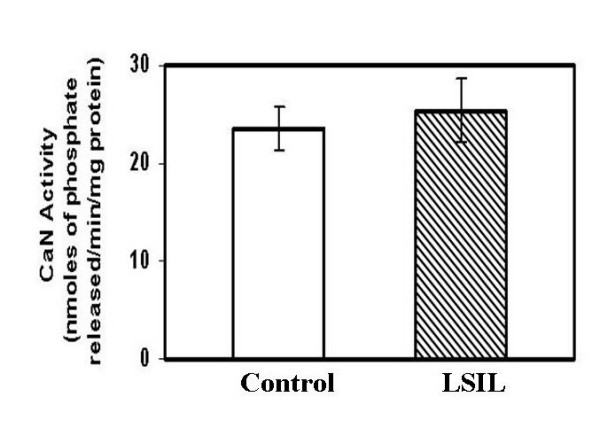
Calcineurin activity as determined in the cervical scrapes obtained from control (N = 25) and those diagnosed with low-grade intraepithelial squamous lesion (LSIL) or CIN1. Note, no significant change in calcineurin activity in control and LSIL group.

To correlate the decrease in calcineurin activity with the early dysplastic changes, we collected cervical scrapes from thirty women attending the outpatient department in the hospital. Of these four were diagnosed as Low-grade squamous intraepithelial lesions (LSIL) by cytology and the rest were cytologically normal but with chronic inflammation. We didn't observe any significant change in the calcineurin activity between the LSIL and the controls in the cervical scrapes (Fig. 4).

### 4. NFAT activity

**Figure 5:**
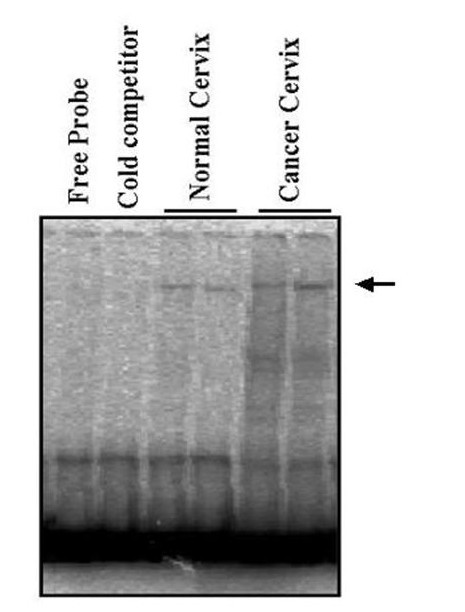
NFAT-DNA-binding activity in normal and cervical cancer nuclear extracts. Briefly 100 μg of nuclear extracts was incubated with ^32^P-labelled NFATc oligonucleotide and Electrophoretic mobility shift assay was performed as described in Material and methods. Lane one contains free probe without nuclear extract and lane 2 contains 100-fold excess of unlabelleled NFAT oligonucleotide as a specific competitor. Specific NFAT- DNA complexes are indicated by arrow.

In view of our observation on downregulation of calcineurin activity in cervical carcinoma, we wanted to investigate if there is any alteration in the DNA-binding activity of NFAT, one of the major substrates of calcineurin. We analyzed the nuclear extracts from 5 squamous cell carcinoma and 5 controls by incubating it with the radiolabelled oligonucleotide probes specific for NFAT binding. Interestingly we didn't find any significant change in the DNA binding affinity of NFAT between cancer and the control samples (Fig. 5).

### 5. HPV typing and testing

A PCR-based reverse line blot assay (40) was used to check the presence of HPV types in the squamous cell carcinomas. We could detect oncogenic HPV-types (16,18,31,45 etc) in all of the cervical cancer but none in the control samples, however we could not correlate the presence of HPV-types with any variation in calcineurin activity (data not shown).

## Discussion

The present study shows downregulation of calcineurin activity in invasive cervical cancer. High calcineurin activity can lead to apoptosis [33], thereby indicating that the downregulation in calcineurin activity can help in cellular survival thereby promoting neoplastic progression. In a previous study [41] a decrease in calcineurin activity in MCF-7 cells following retinoic acid treatment has been linked to downregulation in the expression level of catalytic subunit (A) of calcineurin, however in our study we didn't find any alterations in the level of calcineurin A.

The basis for the decrease in calcineurin activity observed in cervical neoplasia in absence of a change in Calcineurin A content needs to be identified and we speculate that a change in redox state of the cancer cell due to oxidative stress contributes to the alteration in the activity of calcineurin without altering its content. Calcineurin by virtue of its Fe^2+^-Zn^2+ ^binuclear center in its active site is oxidized to Fe^3+^-Zn^2+ ^by superoxide and hydrogen peroxide ions [42]. Superoxide dismutase (SOD), a free radical scavenging enzyme, protects calcineurin from inactivation by superoxide and hydrogen peroxide ions [43,44]. Low levels of the antioxidants such as glutathione, Vitamin E and C as well as superoxide dismutase have been reported to be lower in cervical cancer [45,46], which in turn can modulate the calcineurin activity. Interestingly the lower SOD in circulation reported in cervical cancer [46] may have a role in downregulation of the serum calcineurin activity. There is clearly strong evidence on the involvement of reactive oxygen species (ROS) in cervical cancer [47] and it can most likely alter the calcineurin activity atleast in the tissues without affecting its expression. This argument is also supported by an observation that activity of creatine kinase B, a ROS sensitive protein, is downregulated in cervical cancer [48]. Another possible mechanism of inhibition in calcineurin activity is through upregulation of naturally occurring endogenous inhibitors of calcineurin such as calcipressin 1/DSCR1 and forskolin binding protein [31,49]. The role of these negative regulators of calcineurin in cervical cancer is not known. However it is interesting to note that oxidative stress modulates the calcipressin expression and phosphorylation status [50], which in turn can affect the calcineurin activity.

The natural history of invasive cervical cancer involves sequential progression of persistent HPV infection to reversible LSIL (CIN1) and then to high-grade intraepithelial lesions (HGIL/CIN2). In the presence of other precipitating factors HSIL is a direct precursor of invasive cancer [51]. To evaluate if calcineurin activity is also altered in early precancerous lesions we examined the calcineurin activity in cervical scrapes collected from LSIL cases. Interestingly, there was no significant alteration in the calcineurin activity in the early precursor lesions (LSIL) compared to the control group. None of the samples collected in this study were of HSIL. It has to be however noted that unlike the biopsy specimens of squamous cell carcinoma consisting mostly of the clonally derived malignant cells, the cervical scrapes collected is a mixed population of both the normal and the dysplastic cells. It is possible that the overwhelming number of normal cells in the cervical scrapes diagnosed as LSIL resulted in no significant difference in the calcineurin activity between the control and LSIL group. On the other hand more samples of LSIL and HSIL precursor lesions have to be analyzed before we rule out the possibility of calcineurin involvement in the early stages of cervical cancer progression. Nonetheless, the significant decline in calcineurin activity in invasive squamous cell carcinoma is suggestive of its role in cervical neoplasia.

The regulation of gene expression in response to calcium stimuli is one of the most explored functions of calcineurin. Importantly, the critical target of calcineurin is the NFAT family of transcription factors. Calcineurin dephosphorylates NFAT allowing translocation of this protein into the nucleus [19]. Studies conducted on preadipocyte cell line (3T3-L1) have demonstrated that constitutive expression of NFATc1 can induce cellular transformation providing a direct evidence for the oncogenic potential of the NFATc1 transcription factor [52]. Further, NFAT has also been linked to tumor metastasis [53]. In view of the above evidence, it was of interest to investigate the activation of NFAT in cervical cancer. There was no major difference in DNA binding affinity of NFAT from nuclear extracts obtained from cancer and the control samples. The results on the unaltered DNA binding activity of NFAT seem at odds in view of the reduced calcineurin activity in the cervical neoplasia. However, it is possible that regulation of NFAT by calcineurin is cell type specific and is not affected by calcineurin in cervical cells. Another possibility could be, that calcineurin is known to exist in different isoforms (α,β, ↓) and it is probable that the isoforms existing in cervix has a different substrate specificity. Besides NFAT, calcineurin can also modulate the phosphorylation status of other key regulatory proteins such as Bad, IkB and NFkB, which in turn can promote neoplasia by disturbing the balance existing between cell death and proliferation [30,54]. Already the NFkB mediated pathway is known to be upregulated in cervical cancer progression [55]. Decrease in calcineurin activity could lead to cellular survival by deregulating these major players thereby maintaining the malignant phenotype of cervical neoplasia.

## Conclusion

In conclusion, our pilot study is indicative of a possible role of calcineurin-mediated pathway in pathogenesis of cervical neoplasia. While NFAT is a major target of calcineurin, our study rules out the involvement of this transcriptional factor indicating presence of a NFAT independent calcineurin pathway atleast in cervical cancer. The present investigation has opened up new avenues to evaluate further the role of calcineurin in cervical cancer progression.

## Competing interests

The author(s) declare that they have no competing interests.

## Authors' contributions

SP carried out the calcineurin assays and data compilation. HPV testing and typing in cancer cervix samples was carried out by APS. Both SP and APS performed NFAT assay. The samples were provided by URP, MJ and BNR. The study was conceived and coordinated by GR. The manuscript was finalized by GR and approved by all the investigators.

## References

[B1] Franco EL, Schlecht NF, Saslow D (2003). The epidemiology of cervical cancer. Cancer J.

[B2] Bosch FX, de Sanjose S (2003). Human papillomavirus and cervical cancer-burden and assessment of causality. J Natl Cancer Inst Monogr.

[B3] Shanta V, Krishnamurthi S, Gajalakshmi CK, Swaminathan R, Ravichandran K (2000). Epidemiology of cancer of the cervix: global and national perspective. J Indian Med Assoc.

[B4] Pal SK, Mittal B (2004). Improving cancer care in India: prospects and challenges. Asian Pac J Cancer Prev.

[B5] Castellsague X, Bosch FX, Munoz N (2002). Environmental co-factors in HPV carcinogenesis. Virus Res.

[B6] Wang SS, Hildesheim A (2003). Viral and host factors in human papillomavirus persistence and progression. J Natl Cancer Inst Monogr.

[B7] zur Hausen H (1999). Papillomaviruses in human cancers. Proc Assoc Am Physicians.

[B8] Chakrabarti O, Veeraraghavalu K, Tergaonkar V, Liu Y, Androphy EJ, Stanley MA, Krishna S (2004). Human papillomavirus type 16 E6 amino acid 83 variants enhance E6-mediated MAPK signaling and differentially regulate tumorigenesis by notch signaling and oncogenic Ras. J Virol.

[B9] Ma YY, Wei SJ, Lin YC, Lung JC, Chang TC, Whang-Peng J, Liu JM, Yang DM, Yang WK, Shen CY (2000). PIK3CA as an oncogene in cervical cancer. Oncogene.

[B10] Liu B, Fang M, Lu Y, Lu Y, Mills GB, Fan Z (2001). Involvement of JNK-mediated pathway in EGF-mediated protection against paclitaxel-induced apoptosis in SiHa human cervical cancer cells. Br J Cancer.

[B11] Cheung TH, Yu MM, Lo KW, Yim SF, Chung TK, Wong YF (2001). Alteration of cyclin D1 and CDK4 gene in carcinoma of uterine cervix. Cancer Lett.

[B12] Skomedal H, Kristensen GB, Lie AK, Holm R (1999). Aberrant expression of the cell cycle associated proteins TP53, MDM2, p21, p27, cdk4, cyclin D1, RB, and EGFR in cervical carcinomas. Gynecol Oncol.

[B13] Hashiguchi Y, Tsuda H, Nishimura S, Inoue T, Kawamura N, Yamamoto K (2004). Relationship between HPV typing and the status of G2 cell cycle regulators in cervical neoplasia. Oncol Rep.

[B14] Chavez-Blanco A, Perez-Sanchez V, Gonzalez-Fierro A, Vela-Chavez T, Candelaria M, Cetina L, Vidal S, Duenas-Gonzalez A (2004). HER2 expression in cervical cancer as a potential therapeutic target. BMC Cancer.

[B15] Shen MR, Chou CY, Hsu KF, Ellory JC (2002). Osmotic shrinkage of human cervical cancer cells induces an extracellular Cl-dependent nonselective cation channel, which requires p38 MAPK. J Biol Chem.

[B16] Naeger LK, Goodwin EC, Hwang ES, DeFilippis RA, Zhang H, DiMaio D (1999). Bovine papillomavirus E2 protein activates a complex growth-inhibitory program in p53-negative HT-3 cervical carcinoma cells that includes repression of cyclin A and cdc25A phosphatase genes and accumulation of hypophosphorylated retinoblastoma protein. Cell Growth Differ.

[B17] Cheung TH, Lo KW, Yim SF, Chan LK, Heung MS, Chan CS, Cheung AY, Chung TK, Wong YF (2004). Epigenetic and genetic alternation of PTEN in cervical neoplasm. Gynecol Oncol.

[B18] Narayan G, Pulido HA, Koul S, Lu XY, Harris CP, Yeh YA, Vargas H, Posso H, Terry MB, Gissmann L, Schneider A, Mansukhani M, Rao PH, Murty VV (2003). Genetic analysis identifies putative tumor suppressor sites at 2q35-q36.1 and 2q36.3-q37.1 involved in cervical cancer progression. Oncogene.

[B19] Rusnak F, Mertz P (2000). Calcineurin form and function. Physiol Rev.

[B20] Im SH, Rao A (2004). Activation and deactivation of gene expression by Ca2+/calcineurin-NFAT-mediated signaling. Mol Cells.

[B21] Graef IA, Chen F, Chen L, Kuo A, Crabtree GR (2001). Signals transduced by Ca[2+]/calcineurin and NFATc3/c4 pattern the developing vasculature. Cell.

[B22] Lambrechts D, Carmeliet P (2004). Sculpting heart valves with NFATc and VEGF. Cell.

[B23] Schulz RA, Yutzey KE (2004). Calcineurin signaling and NFAT activation in cardiovascular and skeletal muscle development. Dev Biol.

[B24] Lee JI, Ahnn J (2004). Calcineurin in animal behavior. Mol Cells.

[B25] Sugiura R, Sio SO, Shuntoh H, Kuno T (2001). Molecular genetic analysis of the calcineurin signaling pathways. Cell Mol Life Sci.

[B26] Mansuy IM (2003). Calcineurin in memory and bidirectional plasticity. Biochem Biophys Res Commun.

[B27] Molkentin JD, Lu JR, Antos CL, Markham B, Richardson J, Robbins J, Grant SR, Olson EN (1998). A calcineurin-dependent transcriptional pathway for cardiac hypertrophy. Cell.

[B28] Ladner CJ, Czech J, Maurice J, Lorens SA, Lee JM (1996). Reduction of calcineurin enzymatic activity in Alzheimer's disease: correlation with neuropathologic changes. J Neuropathol Exp Neurol.

[B29] Miyakawa T, Leiter LM, Gerber DJ, Gainetdinov RR, Sotnikova TD, Zeng H, Caron MG, Tonegawa S (2003). Conditional calcineurin knockout mice exhibit multiple abnormal behaviors related to schizophrenia. Proc Natl Acad Sci U S A.

[B30] Gooch JL, Pergola PE, Guler RL, Abboud HE, Barnes JL (2004). Differential expression of calcineurin A isoforms in the diabetic kidney. J Am Soc Nephrol.

[B31] Fuentes JJ, Genesca L, Kingsbury TJ, Cunningham KW, Perez-Riba M, Estivill X, de la Luna S (2000). DCR1, overexpressed in Down syndrome, is an inhibitor of calcineurin-mediated signaling pathways. Hum Mol Genet.

[B32] Lian Q, Ladner CJ, Magnuson D, Lee JM (2001). Selective changes of calcineurin (protein phosphatase 2B) activity in Alzheimer's disease cerebral cortex. Exp Neuro.

[B33] Asai A, Qiu J, Narita Y, Chi S, Saito N, Shinoura N, Hamada H, Kuchino Y, Kirino T (1999). High level calcineurin activity predisposes neuronal cells to apoptosis. J Biol Chem.

[B34] Wang HG, Pathan N, Ethell IM, Krajewski S, Yamaguchi Y, Shibasaki F, McKeon F, Bobo T, Franke TF, Reed JC (1999). Ca2+-induced apoptosis through calcineurin dephosphorylation of BAD. Science.

[B35] Baksh S, DeCaprio JA, Burakoff SJ (2000). Calcineurin regulation of the mammalian G0/G1 checkpoint element, cyclin dependent kinase 4. Oncogene.

[B36] Kahl CR, Means AR (2003). Regulation of cell cycle progression by calcium/calmodulin dependent pathways. Endocr Rev.

[B37] Andre N, Roquelaure B, Conrath J (2004). Molecular effects of cyclosporine and oncogenesis: a new model. Med Hypotheses.

[B38] Hojo M, Morimoto T, Maluccio M, Asano T, Morimoto K, Lagman M, Shimbo T, Suthanthiran M (1999). Cyclosporine induces cancer progression by a cell-autonomous mechanism. Nature.

[B39] Padma S, Subramanyam C (2002). Clinical significance of serum calcineurin in acute leukemia. Clin Chim Acta.

[B40] Gravitt PE, Peyton CL, Apple RJ, Wheeler CM (1998). Genotyping of 27 human papillomavirus types by using L1 consensus PCR products by a single-hybridization, reverse line blot detection method. J Clin Microbiol.

[B41] Sanli UA, Uslu R, Karabulut B, Sezgin C, Selvi N, Aydin HH, Saydam G, Goker E, Omay SB (2003). Alterations in the activity and expression of serine/threonine protein phosphatases during all trans retinoic acid-induced apoptosis in breast cancer cells. Oncol Rep.

[B42] Namgaladze D, Hofer HW, Ullrich V (2002). Redox control of calcineurin by targeting the binuclear Fe^2+^-Zn^2+ ^center at the enzyme active site. J Biol Chem.

[B43] Ullrich V, Namgaladze D, Frein D (2003). Superoxide as inhibitor of calcineurin and mediator of redox regulation. Toxicol Lett.

[B44] Wang X, Culotta VC, Klee CB (1996). Superoxide dismutase protects calcineurin from inactivation. Nature.

[B45] Ahmed MI, Fayed ST, Hossein H, Tash FM (1999). Lipid peroxidation and antioxidant status in human cervical carcinoma. Dis Markers.

[B46] Manju V, Balasubramanian V, Nalini N (2002). Oxidative stress and tumor markers in cervical cancer patients. J Biochem Mol Biol Biophys.

[B47] Giuliano A (2003). Cervical carcinogenesis: the role of co-factors and generation of reactive oxygen species. Salud Publica Mex.

[B48] Choi H, Park CS, Kim BG, Cho JW, Park JB, Bae YS, Bae DS (2001). Creatine kinase B is a target molecule of reactive oxygen species in cervical cancer. Mol Cells.

[B49] Shirane M, Nakayama KI (2003). Inherent calcineurin inhibitor FKBP38 targets Bcl-2 to mitochondria and inhibits apoptosis. Nat Cell Biol.

[B50] Lin HY, Michtalik HJ, Zhang S, Andersen TT, Van Riper DA, Davies KK, Ermak G, Petti LM, Nachod S, Narayan AV, Bhatt N, Crawford DR (2003). Oxidative and calcium stress regulate DSCR1 (Adapt78/MCIP1) protein. Free Radic Biol Med.

[B51] Stoler MH, Rohan TE and Shah KV (2004). The pathology of cervical neoplasia. Cervical Cancer: From etiology to prevention.

[B52] Neal JW, Clipstone NA (2003). A constitutively active NFATc1 mutant induces a transformed phenotype in 3T3-L1 fibroblasts. J Biol Chem.

[B53] Jauliac S, Lopez-Rodriguez C, Shaw LM, Brown LF, Rao A, Toker A (2002). The role of NFAT transcription factors in integrin-mediated carcinoma invasion. Nat Cell Biol.

[B54] Steffan NM, Bren GD, Frantz B, Tocci MJ, O'Neill EA, Paya CV (1995). Regulation of IkB alpha phosphorylation by PKC- and Ca^2+^-dependent signal transduction pathways. J Immunol.

[B55] Nair A, Venkatraman M, Maliekal TT, Nair B, Karunagaran D (2003). NF-kappaB is constitutively activated in high-grade squamous intraepithelial lesions and squamous cell carcinomas of the human uterine cervix. Oncogene.

